# *In situ* observation of nanotwins formation through twin terrace growth in pulse electrodeposited Cu films

**DOI:** 10.1038/s41598-017-10096-5

**Published:** 2017-09-29

**Authors:** Gong Cheng, Heng Li, Gaowei Xu, Wei Gai, Le Luo

**Affiliations:** 1 State Key Laboratory of Transducer Technology, Shanghai Institute of Microsystem and Information Technology, Chinese Academy of Sciences (CAS), Shanghai, 200050 China; 2 University of Chinese Academy of Sciences, Beijing, 100049 China

## Abstract

Nanotwinned copper (nt-Cu) shows a broad application prospects as interconnection materials in integrated circuit industry, since it combines the excellent mechanical and electrical properties. However, the formation and growth behavior of twin lamellae in pulse electrodeposited copper films are not fully understood. In this work, a series of electroplated copper films are prepared by verifying the electroplating parameters and the microstructures are analyzed using scanning electron microscope (SEM) and transmission electron microscope (TEM). The surface morphology indicates strong evidence of stacked twin terraces and layers, suggesting that nanotwins grow up layer by layer. Combined with *in situ* characterization by SEM and molecular dynamics (MD) simulation, it is proved that the terraces originate from the triple junction of grain boundaries and grow up by extending along the lateral (111) crystal plane. A twin terrace-growing model for nt-Cu is then deduced, which distinguishes from deformation twins or annealed twins. This growth model would be prospective to help obtain high quality of nt-Cu in industry.

## Introduction

Nanotwinned copper (nt-Cu) was initially reported in 1975^1^, and it draws wide attention since the year 2004 after the recognition of its combined mechanical and electrical properties^2–8^. Benefit from high-density twin boundaries (TBs) and the reaction with dislocations, nt-Cu simultaneously demonstrates ultra-high strength^9^, high ductility^2,10^, superior thermal stability^11–13^, good conductivity^2^, property of eliminating Kirkendall void^14,15^ and enhanced electromigration resistance^16^, which is significantly beneficial to semiconductor industry^3,17–19^. So far, mainly two kinds of electroplated copper films are reported, i.e. the columnar grains with high density of TBs parallel with the film surface^15,20,21^ and equiaxed grains with TBs perpendicular to the film surface (parallel with the growth direction)^22,23^. Previous investigations have emphasized the contribution of current density, grain boundary (GB), and stress to the formation of nt-Cu. The morphology as well as the TB during electroplating is depicted by a bunching mechanism^24^. Skiba proposed a theoretical model that the cooperative emission of partial dislocations from GBs contributes to the formation of deformation nanoscale twins^25^, but there is a big difference between deformation twins and growth twins. Tu *et al*. found periodical stress increasing and relaxation during the formation of nt-Cu in electroplating of Cu film, and a conjecture of recrystallization model was proposed^26^. Liao claimed that the relaxation of coalescence induced stress is responsible for the formation of a high density of TBs^27^. However, no distinct evidence of twins formation process is detected in their studies, and the nucleation and growth behavior of nanoscale twins in the pulse electrodeposited copper film is yet not fully understood.

This paper reports our recent study on the formation process of nanoscale twins by morphology analyses and MD simulation. The whole procedure of nucleation and growth of twins is *in situ* observed by SEM, and the result reveals that the growth twins nucleate at the triple junction of GBs in pulse electrodeposited Cu films, which is consistent with the MD simulation. Furthermore, strong evidence discovered by microstructural analyses indicates that twin lamellae grow up by extending along the flat facet. Then, a twin terrace model is proposed, and this growth model would be prospective to help obtain high quality of nt-Cu in industry.

## Results

### Morphology and film stress of electroplated copper

**Figure 1:**
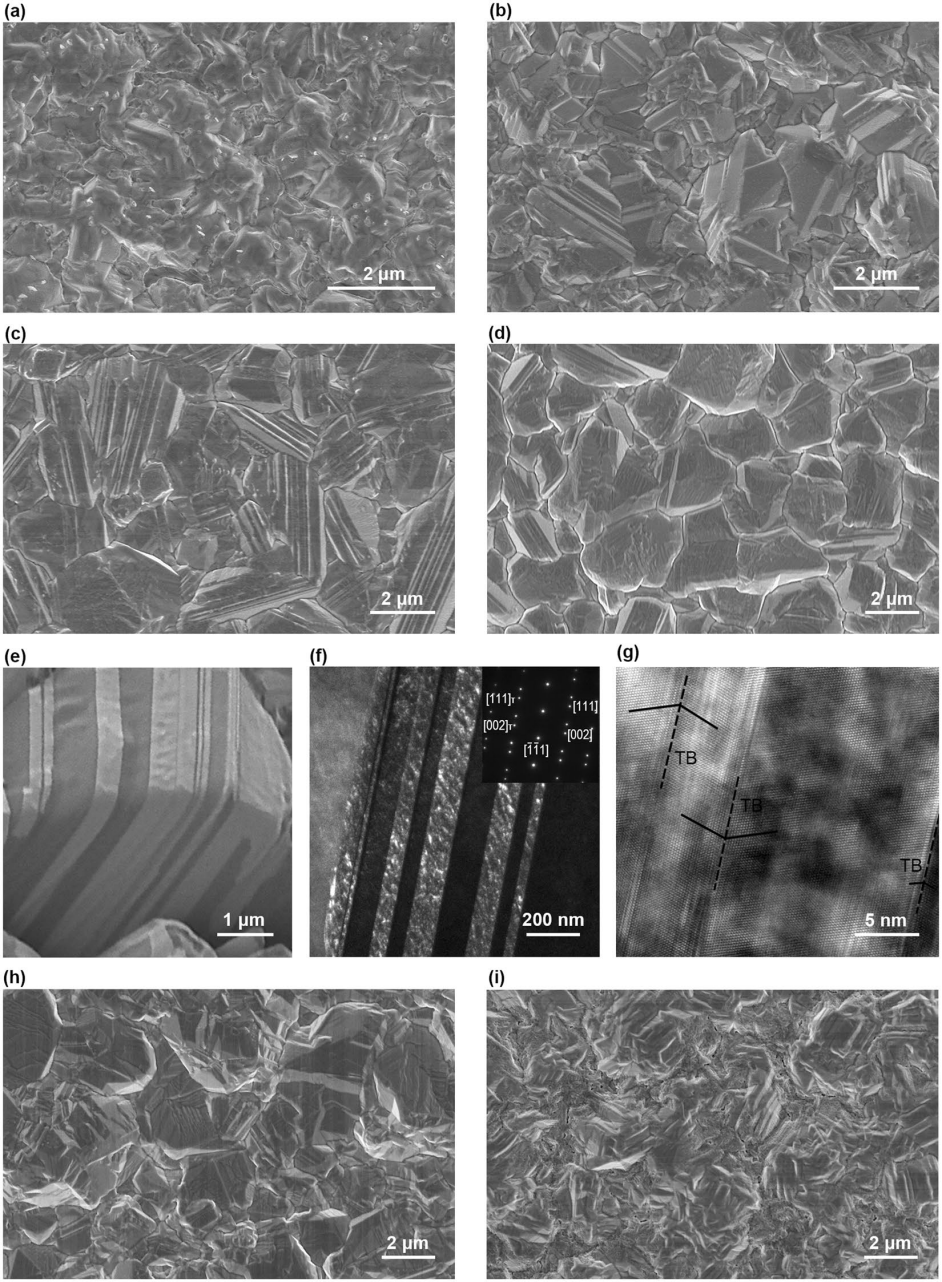
Surface morphology images of as-deposited copper film samples. (**a**–**d**) SEM images of PED-1, PED-6, PED-396, and PED-1196, respectively. (**e**) FIB image of the surface and cross-section of PED-396. (**f**) Cross-sectional TEM dark field image of sample PED-396, with the inset of selected area electron diffraction (SADE) pattern. (**g**) Cross-sectional High-resolution TEM image of sample PED-396. Obvious twins are detected in FIB and TEM images. (**h**,**i**) SEM image of as-deposited DCD-4 and DCD-20, respectively. No terrace-like morphology is detected in DCD prepared samples.

A series of examples using pulse electrodeposition (PED) method with different parameters are prepared. Two sets of direct current deposited (DCD) samples are also prepared for comparison. The thicknesses of electroplated coppers are about 7 μm. Table 1 provides a selected subset of the most representative samples. The entire list of 10 sets can be found in Supplementary Table S1. The average current density (*J*_*avg*_) is calculated from peak current density (*J*_*on*_) and the on-time (*t*_*on*_) and off-time (*t*_*off*_) of the current waveform. Typical surface morphologies of PED-1, PED-6, PED-396, PED-1196, DCD-4, and DCD-20 are shown in Fig. 1a~d and h~i, respectively. The SEM images of other samples can be found as Supplementary Figure S1. The measured film stresses and average grain diameters are also listed in Table 1. As revealed, obvious tensile stress is detected in as-deposited Cu films, and there is a distinct relationship between *t*_*off*_ and film stress. The film stress decreases from 94.9 MPa to 21.8 MPa as *t*_*off*_ increases from 1 ms to 1196 ms, while *t*_*on*_ remains 4 ms. Meanwhile, with the extension of *t*_*off*_, *J*_*avg*_ decreases, and therefore the average grain diameters, which are calculated from more than 300 measured results, increases from 1.15 μm to 2.08 μm.

**Table 1:** Electrodeposition parameters and statistical data about the microstructures of the samples.

Sample	*J*_*on*_ (A/dm^2^)	*t*_*off*_ (ms)	Frequency (Hz)	*J*_*avg*_ (ASD)	Average grain diameter (µm)	Surface Morphology	Terrace density*	Film stress (MPa)
PED-1	100	1	200	80	1.15	terrace-free	—	94.9
PED-6	100	6	100	40	1.17	terrace	30%	84.3
PED-96	100	96	10	4	1.54	terrace	90%	63.6
PED-396	100	396	2.5	1	1.93	terrace	90%	48.1
PED-1196	100	1196	0.83	0.33	2.10	terrace	20%	21.8
DCD-4	4	—	—	4	2.35	flat-top	—	9.23
DCD-20	20	—	—	20	2.40	rugged	—	62.3

*Terrace density is defined as the percentage of terrace-like twinned grain area in the total area.

**The on-time (*t*_*on*_) of pulse electroplated samples is 4 ms.

By comparing the surface morphologies in Fig. 1, obvious terrace-like morphology is detected in PED prepared samples. The terrace density in Table 1 is defined as the percentage of the grain area with terrace in the total area, and the statistical data results from an area of about 3500 μm^2^. A high density of step-like terraces with a flat facet are easily found in PED prepared samples, so long as the *t*_*off*_ of the electrodeposition is between 96 ms and 396 ms. The terrace density reduces with either the further increment or decrement of *t*_*off*_, as indicated in sample PED-1 and PED-1196. The surface of PED-1 is not fully crystallized, since the *J*_*avg*_ is as high as 80 A/dm^2^ (ASD). As for PED-1196, atoms deposited while *t*_*on*_ have sufficient time to find equilibrium positions during *t*_*off*_. Unsurprisingly, no obvious terrace-like morphology is detected in DCD prepared samples, regardless of the current density while electrodeposition. Instead, flat-top or rugged surface is displayed (Fig. 1h and i). Figure 1e is the cross-sectional FIB image of PED-396. Figure 1f and g are the cross-sectional TEM dark field image with the inset of the selected area electron diffraction (SADE) pattern and high-resolution TEM image, respectively. Clear twin lamellae, which are perpendicular to the film surface, are revealed both in FIB and TEM images, indicating that the grain with terrace contains a high density of twins. Obvious TBs are detected in Fig. 1g. Different from annealing twins^28^ and deformation twins^25,29^, the twin boundaries in growth twins are clear and almost no dislocation or stacking fault is detected.

**Figure 2:**
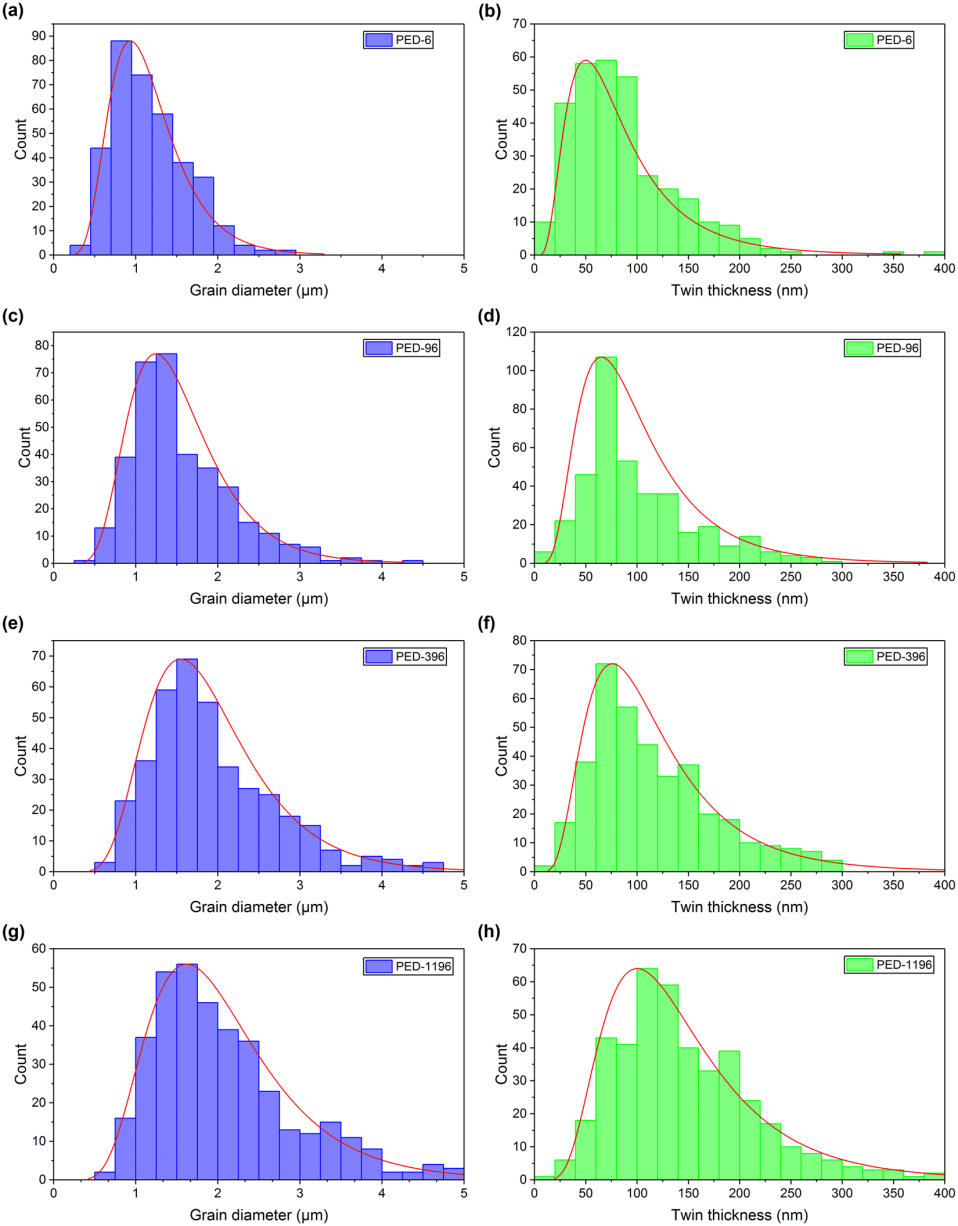
Distribution of grain diameters and twin thicknesses of PED prepared samples. (**a**,**c**,**e**,**g**) The distribution of grain diameter of PED-6, PED-96, PED-396, and PED-1196. Average grain diameters of PED-6, PED-96, PED-396, and PED-1196 are 1.17 μm, 1.54 μm, 1.93 μm, and 2.10 μm, respectively. (**b**,**d**,**f**,**h**) The distribution of twin thickness of PED-6, PED-96, PED-396, and PED-1196. Average twin thicknesses of PED-6, PED-96, PED-396, and PED-1196 are 85.6 nm, 100.7 nm, 116.2 nm, and 145.8 nm, respectively.

The distributions of grain diameter and twin lamellae thickness of PED-6, PED-96, PED-396, and PED-1196 are shown in Fig. 2 after a measurement of more than 300 twins. (For other samples, please refer to Supplementary Figures S2 and S3). According to the statistical data, the average twin lamellae thickness of PED-6, PED-96, PED-396, and PED-1196 are 85.6 nm, 100.7 nm, 116.2 nm, and 145.8 nm, respectively. Similar with grain diameter, the average twin thickness also enlarges along with the extension of *t*_*off*_, because a longer *t*_*off*_ leads to a longer atom diffusion distance and fewer stacking faults.

### *In situ* characterization

**Figure 3:**
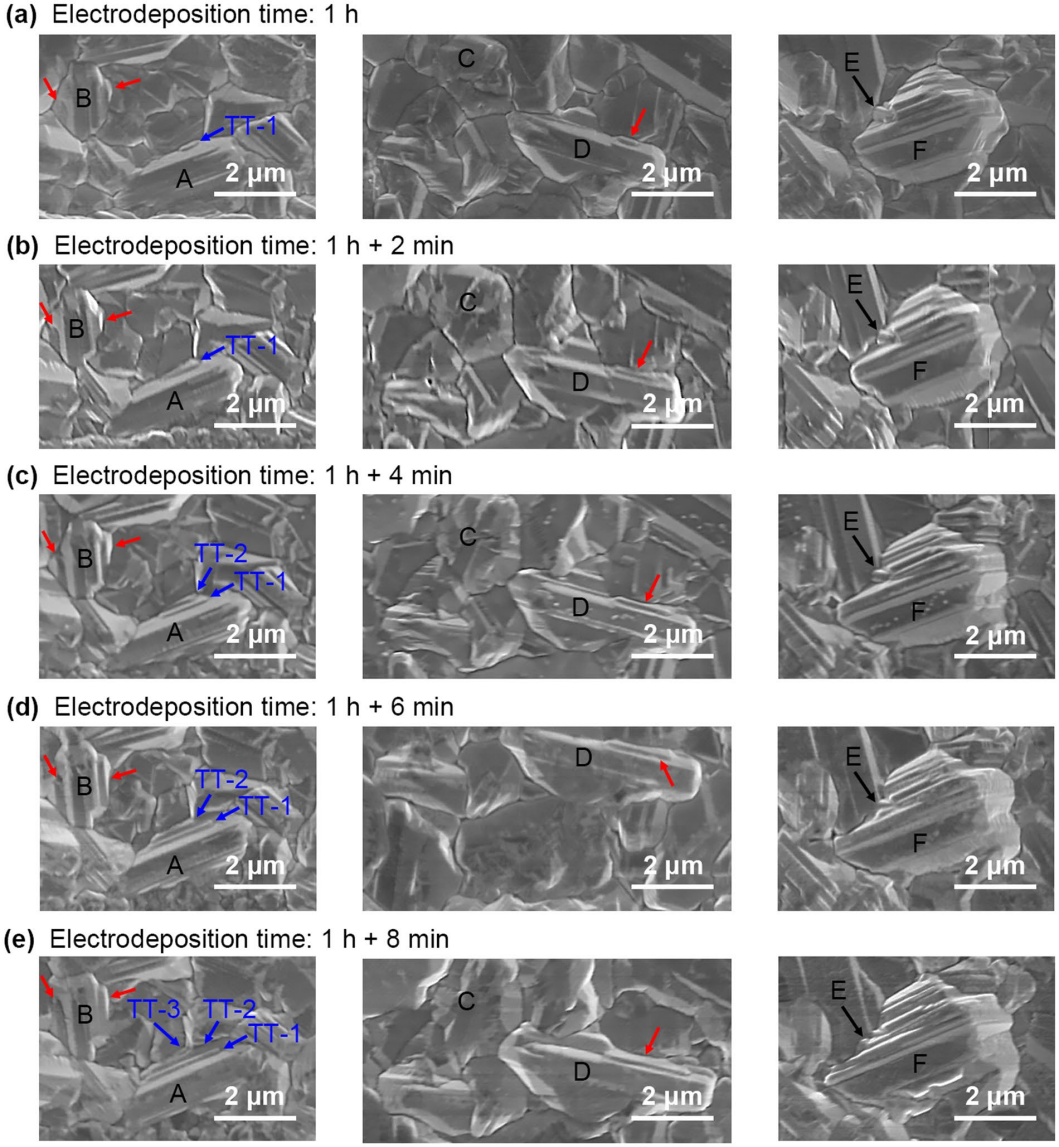
SEM images of *in situ* characterization. (**a**) Surface morphology of PED-96 after electrodeposition for 1 hour. (**b**–**e**) Surface morphology of the same grain, but after another electrodeposition under the same condition for 2 minutes each. Obvious terrace nucleation at the triple junction of GBs and terrace extending along the lateral flat (111) plane is detected. Small grain E gradually coalesces into grain F.

In order to get a complete understanding of the terrace formation process, *in situ* characterization is performed in SEM. The morphology of a certain area of sample PED-96, which is electrodeposited for 1 hour, is characterized by SEM. Then, the morphology of this area is characterized again after another electrodeposition of 2 min, 4 min, 6 min, and 8 min, respectively. As shown in Fig. 3, a series of SEM images reveal the nucleation of twins and terrace growth process. Normal grains indicated as grain C gradually grown into a grain with flat surfaces, once one or more TBs formed inside due to the tensile stress and the extrusion of nearby grains. Besides, the direction parallel with the TB is much easier to prolongation than the perpendicular direction. It is noteworthy that a thickness of approximate 200 nm copper atoms will be deposited after each 2 minutes of pulse electrodeposition, if the atomic deposition is uniform. However, as revealed in Fig. 3, grains with terraces are the grains with preferred origination, and these grains are intended to grow faster than others do. Three typical grains with a high density of twin terraces (TT) marked A, D, and F are shown in Fig. 3. In grain A, triple junction of GBs acts as a successive site of twin nucleation, three twin terraces marked as TT-1~TT-3 are nucleated successively at the triple junction aside the grain A, and grown up during the subsequent electrodeposition process. Several twin terraces (marked by red arrows) located at the triple junctions aside grain B and D are also detected. According to the terrace growth process revealed by Fig. 3, it can be concluded that the terrace grows by extending along the lateral flat (111) plane, until intercepts with the nearby GBs.

There is a tiny grain (grain E) located at the concave corner of grain F, and this tiny grain is thermodynamically stable. However, after several minutes of electrodeposition, this small grain gradually coalesces into grain F. Meanwhile, the terraces are enlarged by more than 1.2 μm after electrodeposition for another 8 minutes. This grain coalescent phenomenon also explains why stress arises in electroplated copper films. Many grain nuclei formed during *t*_*on*_, and some of the small nuclei coalesce together to form larger grains. Thus, the total volume of the grains shrinks due to the disappearance of many GBs and defects^30^. Therefore, the stress of the electrodeposited copper film is always tensile.

### Molecular dynamics simulation

**Figure 4:**
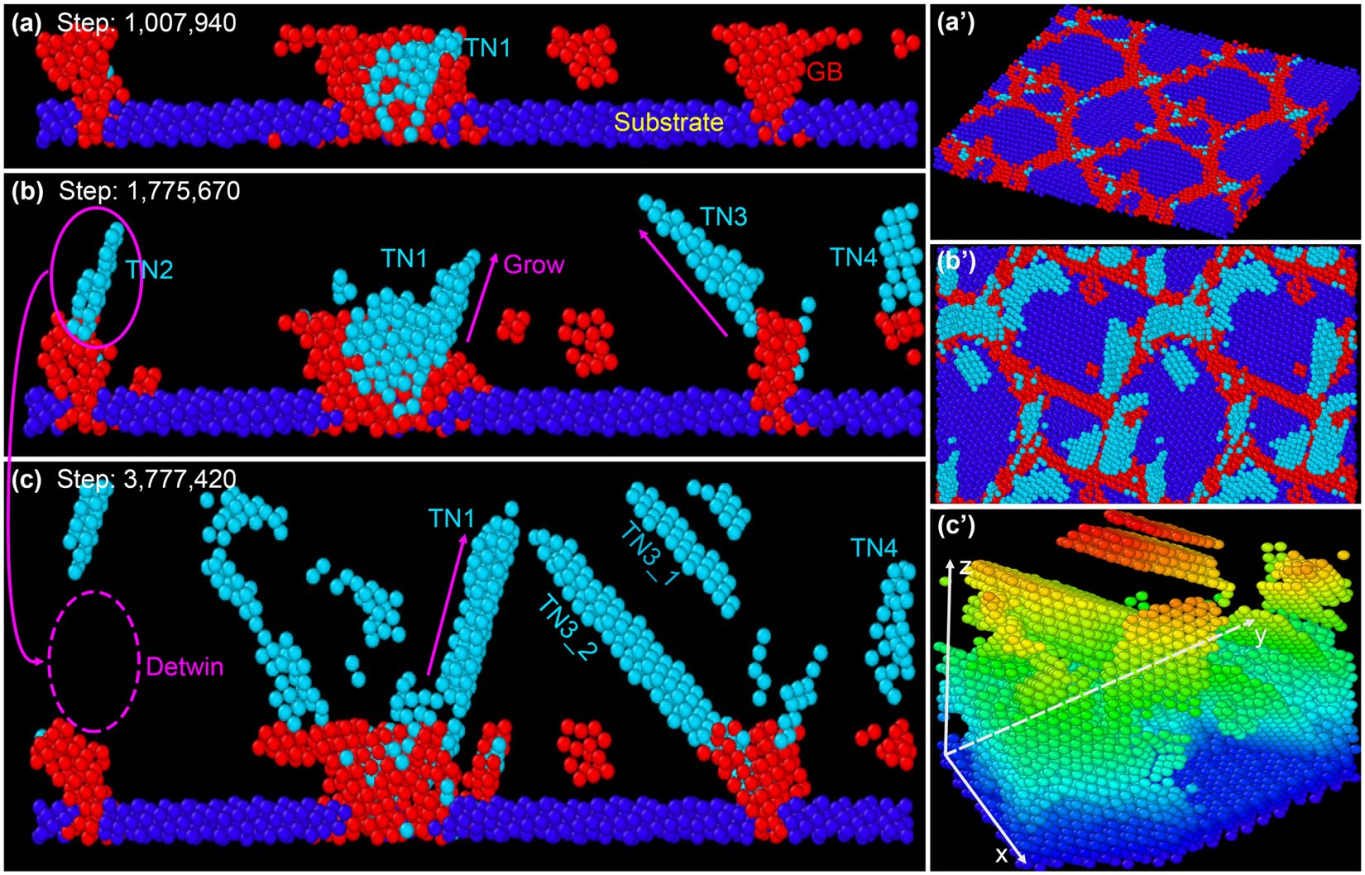
MD simulation of Cu atomic deposition and twin lamellae formation. (**a**–**c**) Cross-section view showing twin nucleation (indicated as TNs) at GBs and grown up at different time steps. (**a’**–**c’**) The corresponding top view or oblique view of (**a**,**b** and **c**). The light blue, red and deep blue stand for hexagonal close-packed lattice (twins), GBs and substrate, respectively.

For a better understanding of the nucleation process, an MD simulation of the Cu deposition on polycrystalline Cu substrate is conducted by Large-scale Atomic/Molecular Massively Parallel Simulator (LAMMPS), as illustrated in Fig. 4. Normal atoms in face-centered cubic lattice are hidden. After about 1 million time steps, twin nucleus (TN) originates from GBs, as marked by TN1 in Fig. 4a. The top view in Fig. 4a’ shows that triple junction of GBs is the initial place for twin lamella nucleation. It is noted that at this time, the twin nuclei are unstable because the diameter of them may be not large enough. Besides, it is found that twin nuclei are formed at several atoms below deposition surface (i.e. the electroplating boundary layer) at the beginning. After another 0.7 million time steps, TN1 grows larger and becomes a stable twin nucleus. Meanwhile, new twin nuclei emerge, indicated as TN2~TN4 in Fig. 4b. Top view in Fig. 4b’ also reveals that many twin nuclei are formed, and mostly located at the triple junction of GBs. At 3.8 million time step, the twin nucleus TN1 further grows larger, while TN2 disappears due to a detwinning process. TN3 splits into two twin lamellae, indicated as TN3-1 and TN3-2. The corresponding oblique view is displayed in Fig. 4c’. This result is consistent well with the experimental results.

It is noted that in the current MD simulated model, most twin lamellae form not directly at the substrate, but several atoms far from the substrate. In fact, it is verified both in our work and in Chen *et al*.’s work^5,20^ that the high density of TBs forms a distance from the film/substrate interface rather than adjacent to the interface. In thick electrodeposited Cu films, an obvious transition layer is found both in the current growth model (refer to Supplementary Figure S4) and the columnar grain growth model with TBs parallel with the films. This is due to the fact that electrodeposition is not an epitaxial growth model, and the transition layer forms because electrodeposited Cu grains are much larger than the magnetron sputtering deposited grains in the seed layer. Moreover, the nt-Cu formation is closely related to atomic flow density. In this model, every 100 steps a single Cu atom is inserted and the deposition rate in the z-direction is 0.3 nm/s, thus the twin nucleation may occur and terrace growth may emerge.

## Discussion

It is known that the rate of grain nuclei formation is directly proportional to the overpotential and current density (atom deposition flux), which results in growing styles varying from continuous growth to terrace growth, and leads to grain morphology varies, as the following formula indicated^31^:

1 $$J={k}_{1}\cdot \exp (\frac{bs{\varepsilon }^{2}}{zekT\eta })$$

where *J* is grain nucleation rate, *k*_1_ is the pre-exponential factor, *b* is the nucleus geometric factor, *s* is the area occupied by one atom on the surface of the cluster, *ε* is the specific edge energy, *z* is atomic chemical valence, *η* is overpotential, *k* and *e* are Boltzmann’s constant and electron charge, respectively. When the flux (average current density) getting larger, a larger amount of grain nuclei will form, leading to a smaller average grain diameter, which is verified by the measured results in Table 1. Meanwhile, larger flux leads to larger intrinsic stress, because the small nuclei or grains intends to coalesce into larger grains and the total volume thus reduced^30^. This process is also *in situ* observed in gain D and E in Fig. 3. In copper films with high stress/strain, the total energy is higher than the strain-relaxed nt-Cu film^26^, because the TB energy is very small, and the ratio of the energy of coherent TB to a high-angle GB is about 0.034 in Cu films^32^. Therefore, higher stress/strain may result in larger probability of twin nucleation.

Grain nucleation only happens during *t*_*on*_ in the pulse electrodeposition process, and stress relaxation and atomic diffusion intend to occur due to the accumulated stress/strain during *t*_*off*_. Periodic *t*_*on*_ and *t*_*off*_ lead to periodic stress increase and decrease^33^. If *t*_*off*_ is short, i.e. PED-1 in our experiment, continuous high flux deposition process gives rise to the incomplete crystallization, and no obvious nanotwins will form. If *t*_*off*_ is long enough for stress relaxation, and Cu atoms able to find equilibrium positions to avoid stacking faults and other defects, low density of twins will be detected just like the case in PED-1196. Only if the electrodeposition parameters such as *J*_*on*_, *t*_*on*_, and *t*_*off*_ are properly adjusted, terrace-like Cu films with high density of nanotwins will be prepared.

**Figure 5:**
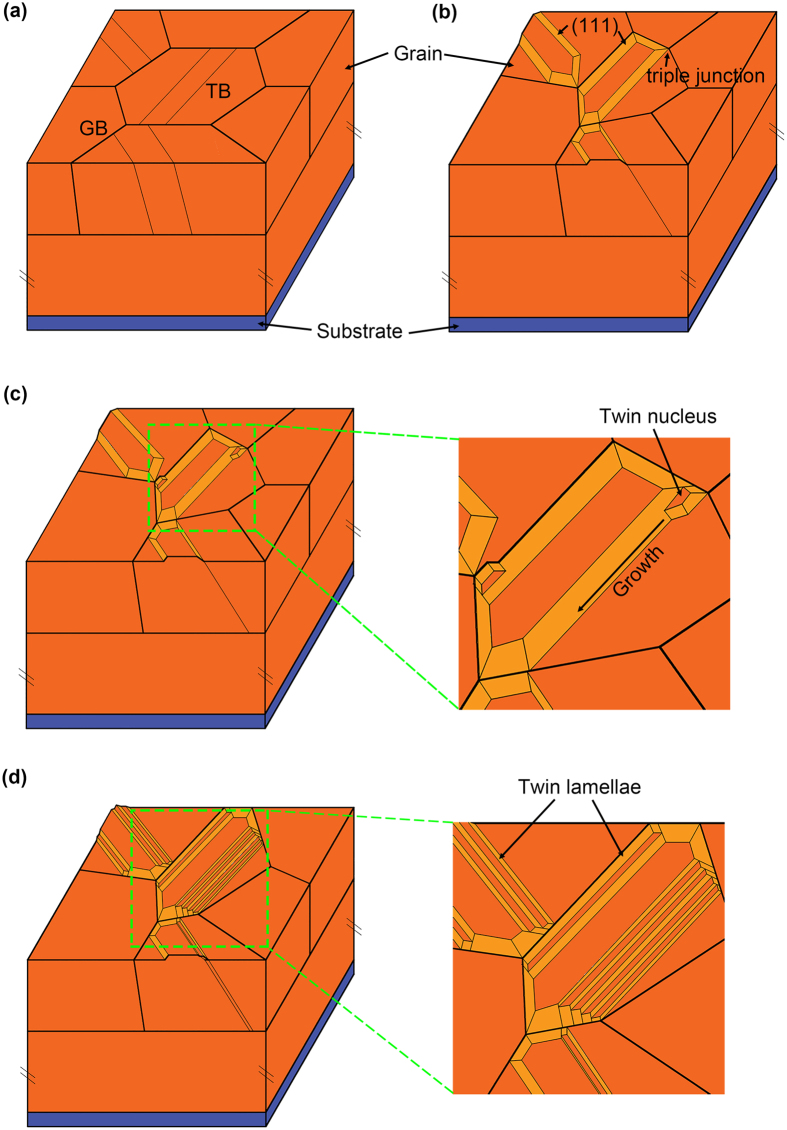
Schematic illustration of nt-Cu formation by twin terrace growth in PED prepared samples. (**a**) TBs form in normal grains due to the tensile stress and the extrusion of nearby grains. (**b**) Grains with TBs inside gradually reveal (111) crystal plane during the subsequent deposition process. (**c**) Twin nucleation originates from the triple junction of GBs. The amplified nucleation site is displayed in the inset. (**d**) Growth of Twin nuclei by extending along the lateral flat (111) plane. Twin terraces form when these lamellae intercept with the opposite GBs. By periodical lateral growth of twin terraces, grains with a high density of twins are formed. The amplified top view morphology is revealed in the inset.

Combine the microstructure analysis and MD simulation, a twin terrace model is proposed to account for the nt-Cu formation by PED method, and the complete process is revealed in Fig. 5. Firstly, TBs form in normal grains due to the tensile stress and the extrusion of nearby grains, and these grains will gradually reveal (111) crystal planes on both the top and lateral surfaces, due to the lowest surface energy, as illustrated in Fig. 5a,b. These grains will act as the grain matrix in the subsequent terrace growth process. Then, twin nuclei originate from the triple junction of GBs (see Fig. 5c), because such an area remains a high level of disorder and therefore with higher residual stress^34,35^. Some twin lamellae with preferred orientation will grow up. When these twin lamellae extend to the nearby grain boundaries, a twin terrace will form, which is in mirror symmetry with the grain matrix about the interface. The twin terrace grows up by laterally extending along the (111) crystal plane, which is similar to the well-known TLK (Terrace-Ledge-Kink) model, except in current situation, the terrace is a twin lamella with several hundred atoms thick. Small current density leads to terrace growing up one by one while large flux results in multi twin terrace growing simultaneously. By periodical twin terrace growth, grains with a high density of nanotwins will form (see Fig. 5d).

Because twins nucleation is a transient process, which is hard to be observed *in situ*, previous literature only gives general statements revolving recrystallization^26^. However, the twins nucleation at GBs is detected in Fig. 3 and verified again by MD simulation. Therefore, it is believed that in thick Cu films, the nt-Cu forms by electrodeposition directly. In addition, it is supported by the fact that the atom diffusion at GB is much faster than in the lattice, and the stress in the triple junction of GBs is much larger than the other places^34^, thus the twins nucleation is easier to occur at the triple junction of GBs.

## Conclusion

In summary, a supposed snapshot of the nanotwins formation is detected by *in situ* observation in SEM and TEM. Combined with the measured stress, a corresponding twin terrace model for nt-Cu formation in PED prepared Cu films is proposed, which consists of three continuous processes. (1) TBs form in normal grains due to the film stress and these grains gradually reveal lateral (111) crystal plane and grow into a matrix for terrace formation. (2) Twin nuclei originate at triple junctions of GBs because of the high level of disorder and high stress. (3) Twin nuclei grow up to form terraces by extending along the lateral (111) crystal plane. Finally, nt-Cu forms by periodical twin terrace nucleation and extension, and the favored twin terraces grow up layer by layer. These findings provide a fundamental understanding of the nucleation and growth process of twin lamellae in pulse electrodeposited Cu films, which complement our previous knowledge about terrace-like growth twins. This will be prospective for industries to obtain a high density of nanotwins in Cu thick films by design atomic deposition flux, which determines twin terrace formation and growth style.

## Methods

### Sample preparations

The Cu films are prepared by PED on 420 μm thick N-type (110) silicon wafer substrate. Firstly, 200 nm thick thermal silicon oxidation is prepared at 1100 °C. Then 100 nm TiW layer is magnetron sputtering deposited as the adhesion layer and diffusional barrier, followed by 500 nm Cu seed layer with a strong <111> preferred orientation. TiW and Cu seed layer are continuously deposited with a pressure of less than 10^−5^ Pa without breaking the vacuum. The 4-inch wafer is diced into an area of 20 × 20 mm^2^ before electroplating. Then the Cu pulse electrodeposition is conducted with the same on-time (*t*_*on*_) of 4 milliseconds (ms) but different off-time (*t*_*off*_) ranging from 1 ms to 1196 ms. The current density *J*_*on*_ during *T*_*on*_ is 100 ASD (A/dm^2^). Phosphorous copper balls are adopted as the soluble anode. The electroplating solution composes of copper sulfate solution and some other additives, in which the concentration of CuSO_4_ and Cl^−^ are 80 g/L and 50ppm, respectively. The pH value of electroplating solution is adjusted to about 1 by H_2_SO_4_. The solution is kept at ambient temperature with mechanically stirring. For comparison, two samples using direct current deposition (DCD) method are also prepared. The thickness of electrodeposited Cu films is about 7 µm. The detailed electroplating parameters of these samples are listed in Table 1 and Supplementary Table S1.

### Microstructure characterizations

The electroplated copper samples are mainly characterized by scanning electron microscopy (SEM, Hitachi S4800) without polish in order to reveal the as-deposited surface morphology. *In situ* characterization of the twin lamellae growth process is performed using SEM. First, a copper film, which is electroplated for an hour under the condition of *t*_*off*_ = 96 ms, is observed in SEM. Then, this copper film is electroplated using the same parameters for another 2 minutes before it is characterized again by SEM, and the same process is repeated for 4 times in order to obtain a successive and complete process of atomic deposition in Cu film. Transmission electron microscopy (TEM, JEM-2100f) is also used to obtain valuable information about TBs. The cross-sectional sample for TEM examination is prepared by focused ion beam (FIB, Quanta 3D FEG 600), and what needs to be emphasized is that the cross section is perpendicular to the twin boundaries. The cross section of the copper film is also characterized using FIB. It is worth noting that a specific angle between the film surface and ion beam direction is needed to obtain a distinct twin contrast in FIB images.

### Film stress measurement

The film stress is calculated using Stoney’s equation^36^ and the curvature of wafer warpage, which is measured by Multi-beam Optical Sensor system. An array of 3 × 4 laser spots with a certain space is projected onto the center of the wafer, and the wafer warpage will be detected by the space change of the reflected beam, which can be monitored by a charge-coupled device (CCD). Based on the principle of laser reflection, the curvature of wafer warpage is detected precisely and the resolution of the facility is 10^−5^ m^−1^.

### Molecular dynamics simulation

The simulation is performed by LAMMPS on three-dimensional polycrystalline Cu substrate containing 3 randomly orientated Voronoi grains. To stand out the GB, the deposition is simulated under a smaller structure with a grain diameter of 3 nm and a total dimension of 7.2 × 7.2 × 7.2 nm^3^. There are no TBs in the substrate. The embedded atom method potential for Cu is adopted. The substrate relaxation is performed at 300 K using a Nose–Hoover thermostat^37^, with the time steps of 7 femtoseconds. Periodic boundary conditions are imposed in all three directions. The substrate is divided into a rigid matrix and surficial active layer. The substrate is relaxed for 500 picoseconds and then changed to a fixed size in the z-direction for deposition. The atom deposition is conducted by DEPOSIT syntax. The temperature of the deposited atoms is controlled by Langevin method^38^. Every 100 steps a single Cu atom is inserted and the deposition rate in the z-direction is 0.3 nm/s.

## Electronic supplementary material

Supplementary information

## References

[1] Merz MD, Dahlgren SD (1975). Tensile-Strength and Work-Hardening of Ultrafine-Grained High-Purity Copper. Journal of Applied Physics.

[2] Lu L, Shen Y, Chen X, Qian L, Lu K (2004). Ultrahigh strength and high electrical conductivity in copper. Science.

[3] Liu CM (2015). Low-temperature direct copper-to-copper bonding enabled by creep on (111) surfaces of nanotwinned Cu. Sci Rep.

[4] Zhao Y, Cheng IC, Kassner ME, Hodge AM (2014). The effect of nanotwins on the corrosion behavior of copper. Acta Materialia.

[5] Liu CM, Lin HW, Lu CL, Chen C (2014). Effect of grain orientations of Cu seed layers on the growth of <111>-oriented nanotwinned Cu. Sci Rep.

[6] Li X, Wei Y, Lu L, Lu K, Gao H (2010). Dislocation nucleation governed softening and maximum strength in nano-twinned metals. Nature.

[7] Zhu YT, Liao XZ, Wu XL (2012). Deformation twinning in nanocrystalline materials. Progress in Materials Science.

[8] Lu K (2016). Stabilizing nanostructures in metals using grain and twin boundary architectures. Nature Reviews Materials.

[9] Zhang L, Zhou H, Qu S (2012). Blocking effect of twin boundaries on partial dislocation emission from void surfaces. Nanoscale research letters.

[10] Lu L, Chen X, Huang X, Lu K (2009). Revealing the maximum strength in nanotwinned copper. Science.

[11] Zhang X, Misra A (2012). Superior thermal stability of coherent twin boundaries in nanotwinned metals. Scripta Materialia.

[12] Niu R (2016). Influence of grain boundary characteristics on thermal stability in nanotwinned copper. Sci Rep.

[13] Zhao YF, Furnish TA, Kassner ME, Hodge AM (2012). Thermal stability of highly nanotwinned copper: The role of grain boundaries and texture. J Mater Res.

[14] Liu TC, Liu CM, Huang YS, Chen C, Tu KN (2013). Eliminate Kirkendall voids in solder reactions on nanotwinned copper. Scripta Materialia.

[15] Hsiao HY (2012). Unidirectional growth of microbumps on (111)-oriented and nanotwinned copper. Science.

[16] Chen KC, Wu WW, Liao CN, Chen LJ, Tu KN (2008). Observation of atomic diffusion at twin-modified grain boundaries in copper. Science.

[17] Tu KN, Hsiao HY, Chen C (2013). Transition from flip chip solder joint to 3D IC microbump: Its effect on microstructure anisotropy. Microelectronics Reliability.

[18] Deng D, Jin Y, Cheng Y, Qi T, Xiao F (2013). Copper nanoparticles: aqueous phase synthesis and conductive films fabrication at low sintering temperature. ACS Applied Materials & Interfaces.

[19] Xu D (2008). Nanotwin formation and its physical properties and effect on reliability of copper interconnects. Microelectronic Engineering.

[20] Liu TC (2012). Fabrication and Characterization of (111)-Oriented and Nanotwinned Cu by Dc Electrodeposition. Crystal Growth & Design.

[21] You ZS, Lu L, Lu K (2011). Tensile behavior of columnar grained Cu with preferentially oriented nanoscale twins. Acta Materialia.

[22] Zhang X (2008). Pulse electroplating of copper film: A study of process and microstructure. Journal of Nanoscience and Nanotechnology.

[23] Hasegawa M (2015). Orientation-controlled nanotwinned copper prepared by electrodeposition. Electrochim Acta.

[24] Pick HJ, Storey GG, Vaughan TB (1960). The structure of electrodeposited copper—I: An experimental study of the growth of copper during electrodeposition. Electrochim Acta.

[25] Ovid’ko IA, Skiba NV (2014). Nanotwins induced by grain boundary deformation processes in nanomaterials. Scripta Materialia.

[26] Xu D (2007). Nanotwin formation in copper thin films by stress/strain relaxation in pulse electrodeposition. Applied Physics Letters.

[27] Chan TC (2014). Growth of large-scale nanotwinned Cu nanowire arrays from anodic aluminum oxide membrane by electrochemical deposition process: controllable nanotwin density and growth orientation with enhanced electrical endurance performance. Nanoscale.

[28] Cheng G, Li H, Zhang W, Xu G, Luo L (2016). Controllable large scaled nanotwin formation in Cu film at lower temperatures. Journal of Physics D: Applied Physics.

[29] Lu L (2009). Stress relaxation and the structure size-dependence of plastic deformation in nanotwinned copper. Acta Materialia.

[30] Kongstein OE, Bertocci U, Stafford GR (2005). *In situ* stress measurements during copper electrodeposition on (111)-textured Au. Journal of the Electrochemical Society.

[31] Paunovic, M. & Schlesinger, M. *Fundamentals of Electrochemical Deposition*. 2nd Edition edn, ISBN: 978-0-471-71221-3 (John Wiley & Sons, 2006).

[32] Anderoglu O, Misra A, Wang H, Zhang X (2008). Thermal stability of sputtered Cu films with nanoscale growth twins. Journal of Applied Physics.

[33] Xu D (2009). *In situ* measurements of stress evolution for nanotwin formation during pulse electrodeposition of copper. Journal of Applied Physics.

[34] Tello J, Bower A, Chason E, Sheldon B (2007). Kinetic Model of Stress Evolution during Coalescence and Growth of Polycrystalline Thin Films. Physical Review Letters.

[35] Chason E (2012). A kinetic analysis of residual stress evolution in polycrystalline thin films. Thin Solid Films.

[36] Stoney GG (1909). The tension of metallic films deposited by electrolysis. P R Soc Lond a-Conta.

[37] Mishin, Y., Mehl, M. J., Papaconstantopoulos, D. A., Voter, A. F. & Kress, J. D. Structural stability and lattice defects in copper: Ab initio, tight-binding, and embedded-atom calculations. *Physical Review B* **63**, doi:10.1103/PhysRevB.63.224106 (2001).

[38] Grønbech-Jensen N, Hayre NR, Farago O (2014). Application of the G-JF discrete-time thermostat for fast and accurate molecular simulations. Computer Physics Communications.

