# Supplementary material for: In situ observation of nanotwins formation through twin terrace growth in pulse electrodeposited Cu films
Source: Sci Rep. 2017 Sep 29;7:12393. doi: 10.1038/s41598-017-10096-5 (PMC5622094; doi:10.1038/s41598-017-10096-5)
Supplement: Supplementary file 1 — Supplementary information [file 41598_2017_10096_MOESM1_ESM.doc]

**Supplementary information**

***In situ* observation of nanotwins formation through twin terrace growth in pulse electrodeposited Cu films**

Gong Cheng^1, 2,*^, Heng Li^1, 2,*^, Gaowei Xu^1,†^, Wei Gai^1^ & Le Luo^1,†^

^1^State Key Laboratory of Transducer Technology, Shanghai Institute of Microsystem and Information Technology, Chinese Academy of Sciences (CAS), Shanghai 200050, China.

^2^University of Chinese Academy of Sciences, Beijing 100049, China.

^*^These authors contributed equally to this work.

^†^Correspondence and requests for materials should be addressed to L.L. (email: leluo@mail.sim.ac.cn) or to G.X. (Email: xugw@mail.sim.ac.cn).


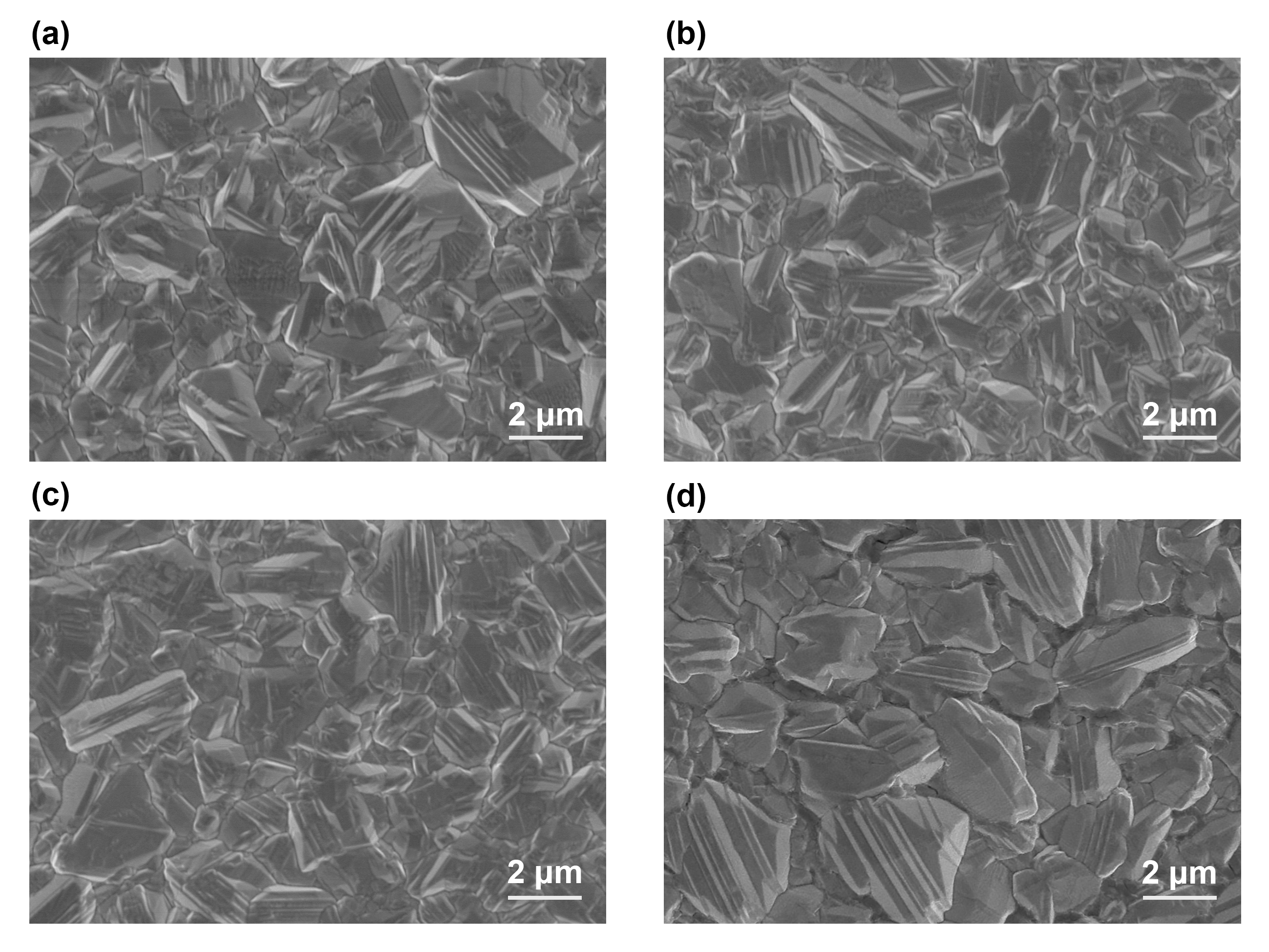


**Supplementary Figure S1** ** SEM images of as-deposited copper film samples. (a)** SEM image of PED-21. Scale bar, 2μm. **(b)** SEM image of PED-46. Scale bar, 2μm. **(c)** SEM image of PED-96. Scale bar, 2μm. **(d)** SEM image of PED-196. Scale bar, 2μm. Obvious terrace-like morphology is found in these samples, though the terrace density varies.


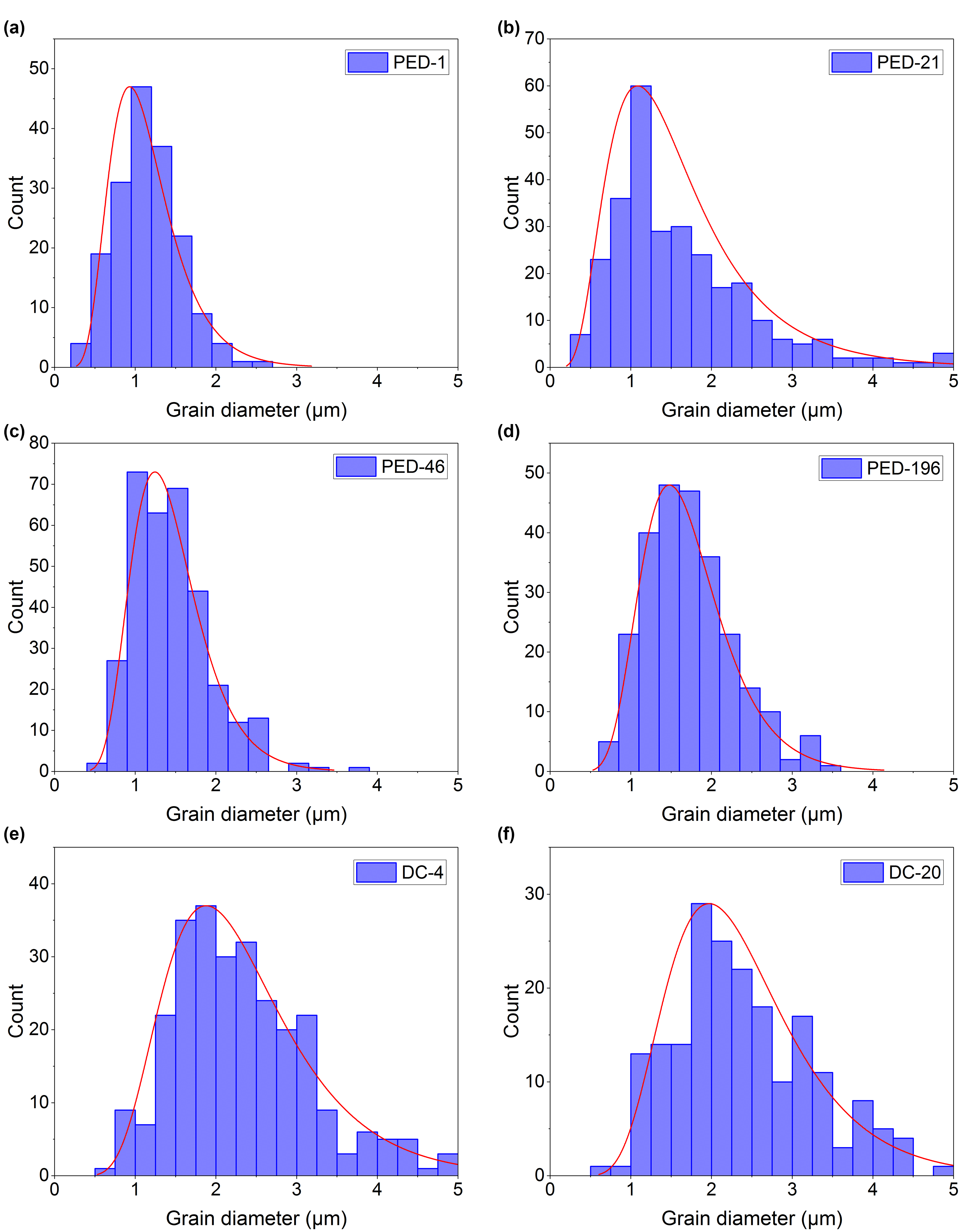


**Supplementary Figure S2  Distributions of grain diameters of PED and DCD prepared samples. (a ~ e)** Distributions of grain diameters of sample PED-1, PED-21, PED-46, PED-196, DCD-4, and DCD-20, respectively. The average grain diameters of **a** ~ **e** are 1.15 μm, 1.61 μm, 1.44 μm, 1.71 μm, 2.35 μm, and 2.40 μm, respectively. In PED prepared samples, the average grain diameter is approximately increasing with the extension of *t_off_*.

**
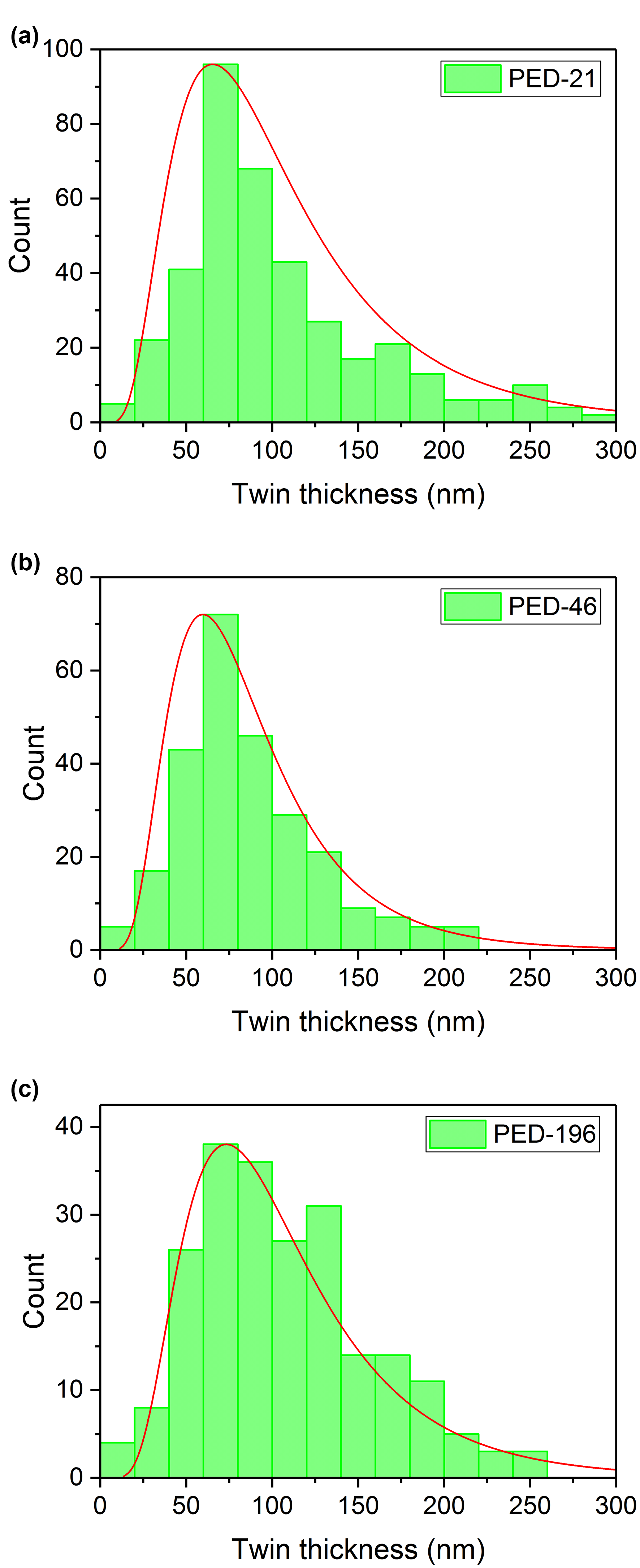
**

**Supplementary Figure S3  Distributions of twin thicknesses of PED prepared samples. (a ~ c)** Distributions of twin thicknesses of sample PED-21, PED-46, and PED-196, respectively. The average twin thicknesses of **a** ~ **c** are 104.7 nm, 86.5 nm, and 107.9 nm, respectively.

**Supplementary Table S1  Electrodeposition parameters and statistical data about the microstructures of the samples.**

| Sample | *J_on_* (A/dm^2^) | *t_off_* (ms) | Frequency（Hz） | *J_avg_* (A/dm^2^) | Average grain diameter (µm) | Surface morphology | Terrace density* | Average twin thickness (nm) | Film stress (MPa) |
| --- | --- | --- | --- | --- | --- | --- | --- | --- | --- |
| PED-1 | 100 | 1 | 200 | 80 | 1.15 | terrace-free | --- | --- | 94.9 |
| PED-6 | 100 | 6 | 100 | 40 | 1.17 | terrace | 30% | 85.6 | 84.3 |
| PED-21 | 100 | 21 | 40 | 16 | 1.61 | terrace | 45% | 104.7 | 75.7 |
| PED-46 | 100 | 46 | 20 | 8 | 1.44 | terrace | 70% | 86.5 | 65.3 |
| PED-96 | 100 | 96 | 10 | 4 | 1.54 | terrace | 90% | 100.7 | 63.6 |
| PED-196 | 100 | 196 | 5 | 2 | 1.71 | terrace | 90% | 107.9 | 38.6 |
| PED-396 | 100 | 396 | 2.5 | 1 | 1.93 | terrace | 90% | 116.2 | 48.1 |
| PED-1196 | 100 | 1196 | 0.83 | 0.33 | 2.10 | terrace | 25% | 145.8 | 21.8 |
| DCD-4 | 4 | --- | --- | 4 | 2.35 | flat-top | --- | --- | 9.23 |
| DCD-20 | 20 | --- | --- | 20 | 2.40 | rugged | --- | --- | 62.3 |

*Terrace density is defined as the percentage of terrace-like twinned grain area in the total area.

** The on-time (*t_on_*) of pules electroplated samples are 4 ms.

**Supplementary Discussion**

Supplementary Figure S1~S3 and Table S1 provides a detailed information about the morphologies of pulse and direct current electrodeposited samples. Terrace-like morphology is found in PED prepared samples, of which the *t_off_* is longer than 1 ms. Besides, there is a clear evidence shows that the terrace density is closely related to the *t_off_* during electrodeposition. The terrace density reduces either the *t_off_* is shorter than 96 ms or longer than 396 ms. A high density of twin terraces is detected in the samples with a proper *t_off_*.

With a longer *t_off_*, the average current density while electrodeposition is lower, thus the average grain diameter increases, and the film stress decreases. Film stress while electrodeposition is believed to have a significant influence on the twins formation, so longer *t_off_* leads to lower film stress. Therefore, with less strain energy, the twinning process is limited, thus the average twin thickness increases, until there is no terrace formed during pulse electrodeposition.


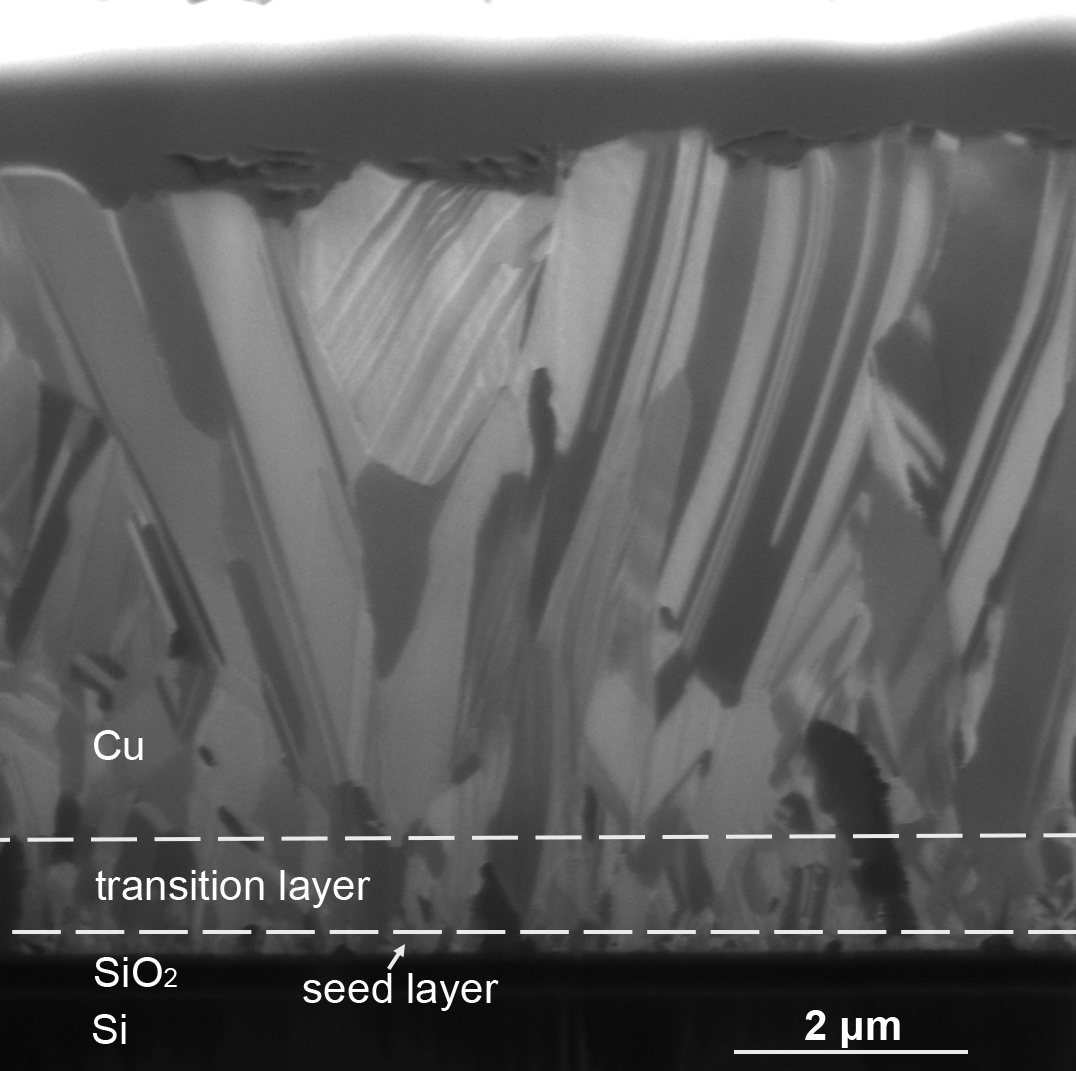


**Supplementary Figure S4  Cross-sectional FIB image of sample PED-396.** The different layers are clearly revealed in this image, and an obvious transition layer is revealed, where the grains are small, and no obvious twins can be detected in the transition layer.
